# Towards the Recognition of Ethiopian Teff (*Eragrostis tef* [Zucc.] Trotter) Flour Under the European Union Geographical Indications System

**DOI:** 10.3390/foods15173080

**Published:** 2026-08-31

**Authors:** Matilde Tura, Rosalba Roccatello, Erica Bensmail, Enrico Valli, Gebreyohannes Girmay, Zenebe Gebreegziabher, Marco Setti, Tullia Gallina Toschi

**Affiliations:** 1 Department of Agricultural and Food Sciences, Alma Mater Studiorum-Università di Bologna, Viale Fanin 40-50, 40127 Bologna, Italy; matilde.tura2@unibo.it (M.T.); rosalba.roccatello@unibo.it (R.R.); erica.bensmail2@unibo.it (E.B.); marco.setti@unibo.it (M.S.); tullia.gallinatoschi@unibo.it (T.G.T.); 2 Interdepartmental Centre for Industrial Agrofood Research, Alma Mater Studiorum-Università di Bologna, Via Quinto Bucci 336, 47521 Cesena, Italy; 3 Department of Agricultural and Food Sciences, Alma Mater Studiorum-Università di Bologna, Piazza Goidanich 60, 47521 Cesena, Italy; 4 Department of Land Resource Management and Environmental Protection, College of Agriculture and Natural Resources, Mekelle University, Mekelle P.O. Box 231, Ethiopia; gebreyohannes.girmay@gmail.com (G.G.); zenebe.gebreegziabher@mu.edu.et (Z.G.)

**Keywords:** Ethiopian teff, geographical indications, food quality, food labelling

## Abstract

This paper explores the potential for promoting Ethiopian teff flour through the European Union’s Geographical Indications (GI) framework. After outlining the evolution of GI regulations in the international trade context and the EU’s role in quality policy for agri-food products, the paper assesses the value of teff as a high-potential product. Originated and domesticated around Axum, teff is a staple crop in Ethiopia, both nutritionally and socio-economically, cultivated by a multitude of smallholder farmers. However, access to European markets is hindered by insufficient enforcement of food safety standards, lack of quality control, and inconsistent labelling practices. Recognition as a GI—specifically as a Protected Geographical Indication (PGI)—could enhance the visibility, traceability, and legal protection of Ethiopia’s teff. The paper also highlights challenges in advocating for the development of a detailed product specification aligned with EU regulations. Integrating teff production into the GI system may strengthen rural economies, preserve traditional knowledge, and increase the international profile of a product with significant nutritional and economic potential.

## 1. Introduction

### 1.1. The Importance of Geographical Indications

Geographical Indications (GIs) are internationally recognized through the 1994 Agreement on Trade-Related Aspects of Intellectual Property Rights (TRIPS), which establishes minimum standards for GI protection. Under this agreement, GIs identify products whose quality, reputation, or other characteristics are essentially attributable to their geographical origin. WTO (World Trade Organization) Members are therefore required to provide legal means to prevent the misleading use of geographical names and acts of unfair competition [1]. The inclusion of GIs within the TRIPS Agreement largely reflected the efforts of the European Union (EU) during the Uruguay Round negotiations, despite strong opposition from several New World countries [2]. Within the EU, the quality policy for agricultural products is based on three main schemes: Protected Designation of Origin (PDO), Protected Geographical Indication (PGI), and Traditional Specialty Guaranteed (TSG), which protect product names linked to geographical origin or traditional know-how (Table 1) [3,4].

**Figure 1:**
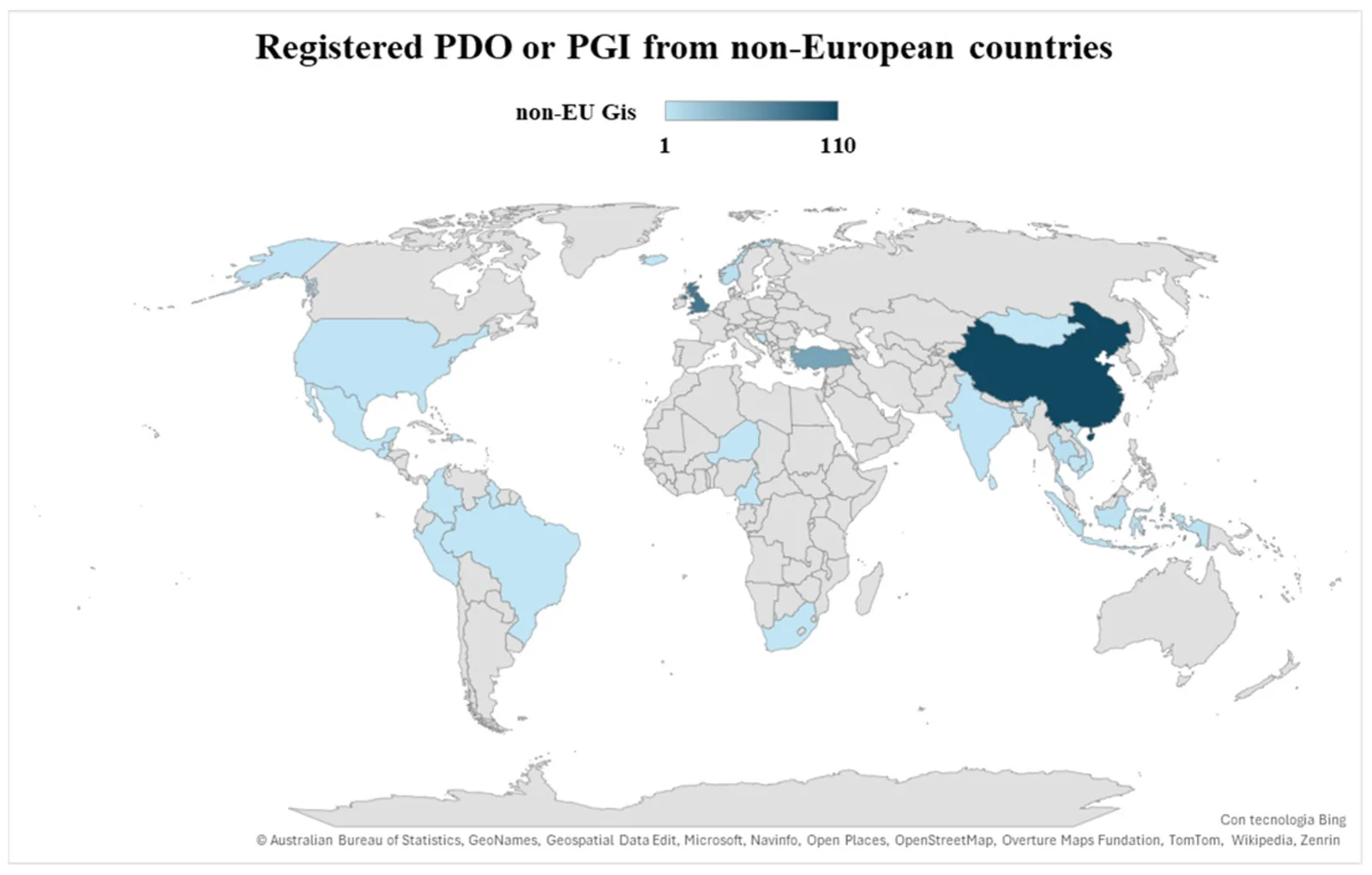
Examples of agricultural products from non-EU countries registered under the EU geographical indications system (figure has been constructed by the extrapolation of data from GI view, https://www.tmdn.org/giview/gi/search, accessed on 20 May 2026).

Nowadays, an increasing number of local agricultural products are being recognized as GIs worldwide, reflecting growing consumer demand for foods whose quality and reputation are closely linked to their place of origin [5]. Beyond Europe, several countries have successfully adopted GI strategies to enhance the market value of traditional agricultural products and support rural development. Well-known examples include Darjeeling tea in India, Kampot pepper in Cambodia, Rooibos tea in South Africa and Colombian coffee, all of which demonstrate how origin-based protection can strengthen product identity, facilitate access to premium international markets and contribute to local economic development (Figure 1) [6,7,8,9].

In parallel with these international developments, Ethiopia has progressively strengthened its intellectual property framework through the Ethiopian Intellectual Property Authority (EIPA), promoting geographical indications, trademarks and the protection of traditional knowledge. Successful experiences such as Ethiopian coffee provide relevant examples of how origin-based protection can enhance product value and international recognition. Since 2004, EIPA has registered trademarks for several regions, i.e., Sidamo, Harar and Yirgacheffe, in over 30 countries under the Ethiopian Coffee Trademarking and Licensing Initiative, demonstrating the potential of origin-based strategies for agricultural exports [10]. More recently, the three-year GOPE (“Geographical indications for origin-linked products in Ethiopia”) project aims to develop a national GI legislative framework and implement a pilot GI in the coffee value chain. These initiatives illustrate Ethiopia’s growing commitment to leveraging geographical indications as tools for rural development, export diversification and the protection of traditional knowledge, providing an institutional foundation that could also support future GI recognition for teff and other traditional agricultural products [11,12]. Since the launch of GOPE, its legislative component has progressed: in October 2025, the Ethiopian Intellectual Property Authority presented a final draft of the national Geographical Indications Law to stakeholders and partner institutions [13]. This development is particularly relevant to any future EU application because, for a geographical indication originating in a third country, Regulation (EU) 2024/1143 requires legal proof that the indication is protected in its country of origin [4].

### 1.2. Requirements for Teff to Enter the EU and the Main Obstacles It Faces

The international GIs experiences are particularly relevant for African countries, where many traditional agricultural products combine strong cultural identity with unique environmental characteristics but still face significant barriers to accessing high-value international markets. Common constraints include insufficient implementation of food safety standards, limited quality control systems, weak traceability mechanisms and inconsistent labeling practices. Strengthening these elements through geographical indication schemes could improve product credibility, facilitate compliance with international market requirements and increase export opportunities [14]. Among these products, Ethiopian teff (*Eragrostis tef* [Zucc.] Trotter), an ancient cereal traditionally domesticated in the Ethiopian highlands, represents a particularly promising candidate because of its strong association with specific production areas, long-standing cultivation traditions and growing international demand. It has a strong link with the geographical location, considering that this region, marked by a cool temperate climate and fertile volcanic soils, provides optimal conditions for its cultivation and has historically supported its agronomic success [15,16]. Teff is also increasingly recognized as one of Ethiopia’s most promising crops for export diversification, owing to its exceptional nutritional profile and rapidly growing international market demand [17,18]. However, realizing this potential requires the development of robust quality standards, traceability systems, and product specifications consistent with EU GI requirements [19]. In addition, the international commercialization of teff has also highlighted important challenges related to intellectual property protection and the recognition of Ethiopia’s traditional knowledge. Despite its growing global popularity, Ethiopia’s access to the European teff market has been historically constrained by intellectual property rights related to teff flour processing. A European patent (EP 1 646 287), granted in 2007 by the European Patent Office (EPO), covered specific technical parameters for teff flour production, including minimum falling number values after grain ripening. Although this patent was subsequently invalidated in The Netherlands (2018) and Germany (2019) on grounds of lack of inventive step, it remained active in several other European countries until its withdrawal in 2020. These legal developments highlighted the limitations of patent systems in protecting traditional knowledge and stimulated broader discussions on alternative intellectual property instruments, including geographical indications, that may better recognize the long-standing relationship between agricultural products, local communities and their territories [20]. Thus, although there is increasing scientific interest in teff, growing international demand for gluten-free cereals and the progressive development of GI frameworks beyond Europe, no study has integrated agronomic, quality, regulatory and institutional evidence to evaluate the feasibility of recognizing Ethiopian teff under the EU GI framework. While existing research has primarily addressed the agronomic, nutritional and socio-economic dimensions of teff, the potential role of GIs as a strategic instrument for protecting its geographical origin, enhancing market value and supporting rural development has received limited attention.

This review therefore asks to what extent the available scientific, regulatory, institutional, and socio-economic evidence supports the recognition of Ethiopian teff flour under the EU PGI scheme, and which gaps would need to be addressed before an application could be credibly pursued. The contribution of the paper lies in integrating bodies of evidence usually considered separately and translating them into requirements for evidence assessment of GI readiness, rather than assuming that nutritional value or cultural importance alone is sufficient to establish eligibility.

Conceptually, the paper adopts an integrative, multidisciplinary evidence-synthesis approach rather than a systematic review. Evidence was considered when it was directly relevant to one or more of these analytical dimensions and to the assessment of PGI eligibility or enabling conditions. The evidence is organized around four analytical dimensions: (i) geographical origin and the link between place and product characteristics or reputation; (ii) grain and flour quality, food safety, and standardization; (iii) production, traceability, and institutional governance; and (iv) socio-economic opportunities, risks, and trade-offs. Peer-reviewed studies are used primarily for scientific and socio-economic evidence, whereas EU legislation, Ethiopian standards, and official institutional documents are used to assess legal and regulatory requirements and the current policy context.

The remainder of the paper is organized as follows: (i) Section 2 examines the historical origin, domestication, genetic diversity, and geographical distribution of Ethiopian teff; (ii) Section 3 reviews grain and flour quality, including origin-related compositional markers, food-safety concerns, and existing flour standards; (iii) Section 4 describes the Ethiopian production system and value chain, with particular attention to household consumption, marketable surplus, post-harvest management, traceability, seed systems, and institutional arrangements; (iv) Section 5 assesses the evidence against the requirements and enabling conditions for EU GI recognition, summarizes the principal gaps and trade-offs, and discusses policy and research priorities.

## 2. Ethiopian Teff: Characteristics Supporting Its Geographical Identity

### 2.1. Teff Origin and History

Historically cultivated in the Ethiopian highlands since before recorded time, teff holds a central place in Ethiopia’s cultural heritage and national identity [21,22]. Teff-based food culture has a very ancient origin, dating back approximately 4000–1000 BC. Teff is considered to have been domesticated by Ethiopian farmers in the areas surrounding Axum and subsequently spread southward, westward, and eastward across Ethiopia, maintaining the diverse landraces in the country over thousands of years that followed [18,23]. The name “teff” originates from the Ethiopian word *teffa*, meaning “lost”, a reference to the extremely small size of the grain, which makes it difficult to recover once dropped [21].

Within its region of origin, Axum teff, locally known as “*taf Hatsebo in Tigrinya*”, is particularly valued and is widely recognized for its dense grain and suitability for different processing methods and food preparations in Ethiopia. Communities in and around Axum developed a wide range of teff-based foods and beverages characterized by well-established fermentation processes and traditional grain-storage practices. These practices represent an important component of the long-standing knowledge associated with the cultivation, processing, and consumption of teff [24].

Teff also occupies an important place in the history of botanical research. Its botanical description was based on plant specimens collected in northern Ethiopia. The botanical nomenclature of teff has undergone several revisions since the eighteenth century. The crop was first described as *Poa tef* Zuccagni in 1775, followed by *Poa abyssinica* Jacquin in 1781, *Eragrostis abyssinica* (Jacq.) Link in 1827, and *Eragrostis pilosa* ssp. *abyssinica* (Jacq.) Asch. and Graeben. in 1900. Its currently accepted botanical name, *Eragrostis tef* (Zucc.) Trotter, was established by Trotter in 1918. This taxonomic history reflects the gradual scientific classification of a crop with a much older history of cultivation in Ethiopia. Although the precise time and location of its domestication remain uncertain, Ethiopia is recognized as the center of origin and diversity of teff, and archaeological evidence points to its domestication in northern Ethiopia during the pre-Axumite period (800–400 BC) [25].

Despite its ancient origin and long history of cultivation in Ethiopia, teff remained relatively little known outside the country for much of its history. Broader international awareness began to increase during the 1960s, when developments in scientific communication and the growing dissemination of research on Ethiopian agriculture, nutrition, and food composition made information on the crop more accessible to the international scientific community (e.g., [26]). Since then, scientific interest in teff has progressively expanded beyond Ethiopia. Throughout its history, teff has been much more than a staple cereal. It has played, and continues to play, an important role in social rituals, religious ceremonies, food traditions, and economic activities. Its long association with farming communities, particularly in northern Ethiopia and around Axum, together with the diversity of traditional foods, beverages, landraces, and processing practices developed over time, reflects its broad cultural, historical, and societal importance in Ethiopia [27].

### 2.2. Geographical Area of Cultivation

Ethiopia is a landlocked country in the Horn of Africa that occupies a total area of 1.2 million square kilometers (420,000 square miles), and its principal natural resource is its arable land, of which 35.68% is farmed at present. The diverse agro-ecological zones of the country, enriched with varied soil fauna and flora, make Ethiopia a center of origin and diversity for several economically significant crops, most notably teff [28,29].

**Figure 2:**
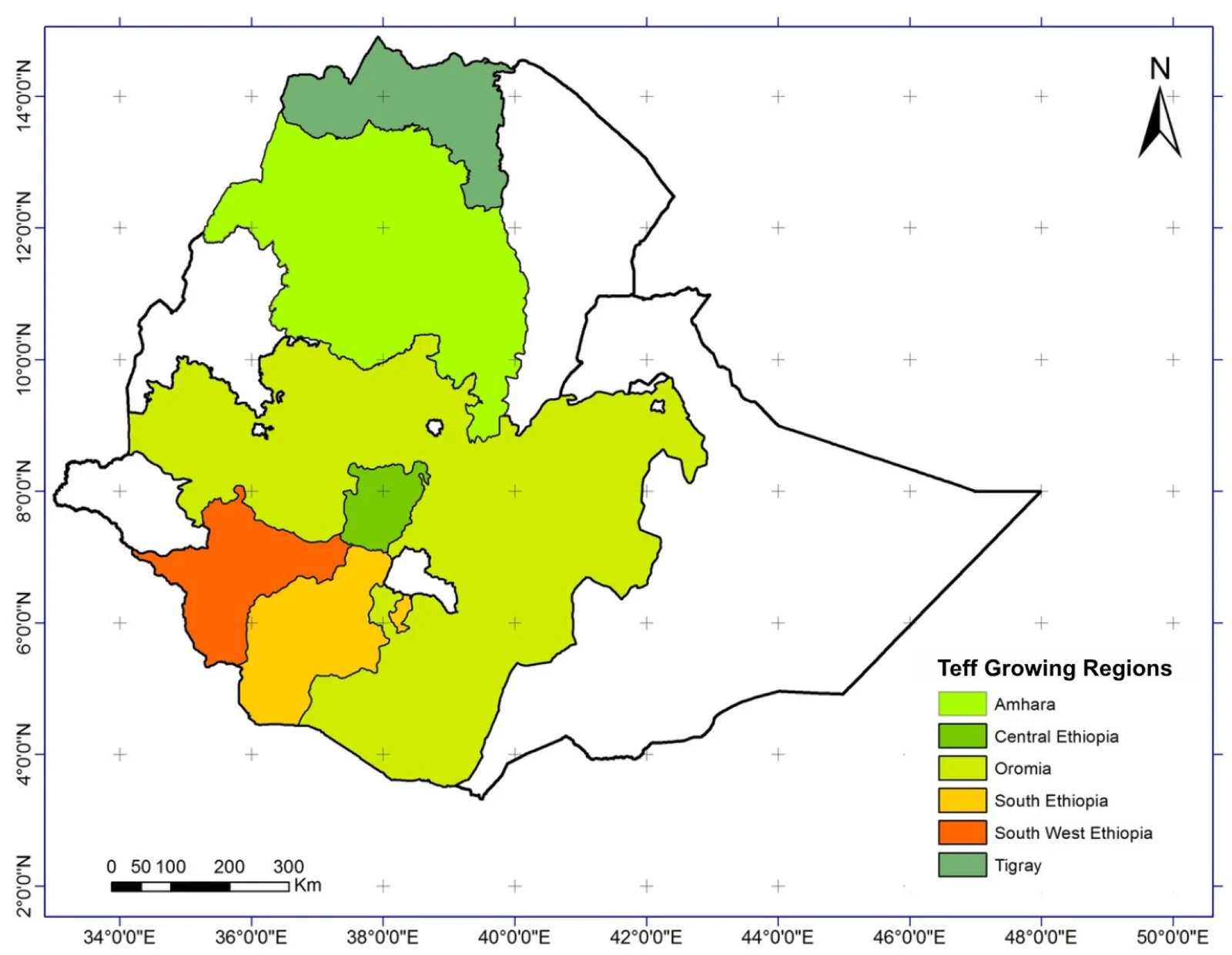
Geographical area in which Teff is cultivated in Ethiopia.

Geographically, teff is mainly cultivated in Ethiopia’s central and northwestern highlands, specifically within the Oromia, Amhara, Southern Nations, Nationalities, and Peoples (SNNP), and Tigray regions [30], as illustrated in Figure 2. The Oromia and Amhara regions dominate, accounting for 85.5% of the total cultivated area and 87.8% of national production [17,31]. Within these, East and West Gojjam (Amhara) and East and West Shoa (Oromia) are key production zones, with East Gojjam alone contributing over 10% of the national annual teff output [17,22]. Smaller regions, such as Tigray and SNNP, contribute 6% and 9.6% of the cultivated area and national production, respectively [17,32].

According to FAO (Food and Agriculture Organization) soil classification and regional statistics, nine major soil types exist in Ethiopia, distinguished by their origin and formation processes. These include Andosols, Vertisols, Cambisols, Regosols, Luvisols, Phaeozems, and Fluvisols, all of which are associated with high agricultural potential. Teff tends to perform better—both in yield and protein content—on Vertisols compared to Andosols [22]. Vertisols, which dominate teff cultivation areas, are characterized by their dark color, high montmorillonite clay content, and richness in magnesium and calcium. Their high clay composition gives them distinctive swelling and shrinking behavior. However, nutrient limitations exist; for example, zinc bioavailability has been identified as a yield-limiting factor in Vertisols [33], while phosphorus and nitrogen are key constraints in dryland production systems [16].

As the center of origin and domestication of teff, Ethiopia holds most of the genetic resources of the crop in the form of landraces, which are characterized by high genetic diversity and adaptability to changing environmental conditions [21,34]. Several varieties are cultivated across the country, each suited to specific environmental and soil conditions [22]. For example, teff grown on black and brown soils generally produces brighter grain color compared to that grown on red soils.

The teff cultivars currently available have been developed through generations of farmer-led selection [29]. Teff is well adapted to the diverse agroecological conditions of Ethiopia, where it is cultivated across both highland and lowland areas, including drought-prone and waterlogged environments, and on a wide range of soil types. It is mainly grown at altitudes between 1500 and 3000 m, under average annual rainfall of 500–1200 mm and temperatures ranging from 15 to 25 °C. Teff performs best in heavy clay soils with a pH between 6.2 and 8.5. Teff is a long-day crop requiring extended exposure to solar radiation, indicating that day length is more critical than light intensity for its growth [21]. Regions in Ethiopia where teff has been cultivated for centuries offer abundant light, which is essential for several vital physiological processes including seed germination, leaf expansion, stem and shoot growth, tillering, flowering, fruiting, and root development. This photoperiod sensitivity presents challenges when cultivating teff outside its native range, where suboptimal light conditions may impair its physiological functions [21]. These factors, along with teff’s physiological plasticity, enable it to thrive in 10 out of the 22 agroecological zones of Ethiopia [35,36].

## 3. Ethiopian Teff: Quality

### 3.1. Teff Quality

Although modern breeding efforts have improved yields, teff has been cultivated in Ethiopia for more than 600 years with minimal use of chemical inputs, and traditional agronomic practices are still widely employed to preserve grain quality. However, advances in crop improvement may also negatively affect nutritional quality through a dilution effect [21]. It is therefore likely that the observed variation in teff nutrient content across Ethiopia is driven more by environmental differences (such as climate and soil conditions) than by genetic factors [37]. Fatty acid profiles are increasingly used to assess the quality and geographic origin of teff. The Amhara region, particularly the East and West Gojjam zones, is one of the leading teff-producing areas of the country. Within this region, the Bichena district in East Gojjam is especially known for producing highly preferred and expensive teff. However, the problem of market fraud—where lower-quality teff is mislabelled and sold as Bichena teff—has raised concerns. This has led to a growing demand for reliable methods to authenticate teff origin. Recent studies have identified specific fatty acids—stearic acid, linoleic acid, oleic acid, and palmitic acid—as effective biomarkers for distinguishing teff according to production zones. Among these, stearic acid has proven to be a particularly useful discriminant marker for teff grown in West Gojjam [38]. In addition, to assess the potential of chemical markers for geographical authentication, a study [38] analyzed the fatty-acid profiles of 72 teff samples collected from three production zones in northwestern Ethiopia using gas chromatography–mass spectrometry. Thirty fatty acids were detected, and multivariate statistical analyses identified compositional patterns capable of discriminating samples by production zone. Stearic acid was identified as a particularly relevant marker for distinguishing teff originating from West Gojjam. These findings provide preliminary evidence that fatty-acid profiling could complement documentary traceability in a future GI control system. However, validation using independent samples, multiple harvest years, additional varieties and wider geographical coverage would be necessary before this approach could be adopted for official certification purposes.

Teff is considered a relatively pest-resistant crop, as its seeds can remain viable for years without moisture and, due to their tiny size, seeds are naturally resistant to weevils and other storage pests. This natural resistance, together with the crop’s resilience to environmental stresses, further reduces the need for chemical treatments. Traditional Ethiopian storage methods, often in humid conditions and in direct contact with soil, can promote fungal growth and mycotoxin contamination, leading to food spoilage. Fungal contamination by Fusarium and Aspergillus has been reported during both cultivation and storage, posing food safety risks due to their potential to produce mycotoxins. Limited studies have detected aflatoxin B1 (AFB1) and ochratoxin A (OTA) in teff, with average levels exceeding the EU regulatory limits by threefold for AFB1 and sevenfold for OTA.

Teff contamination with heavy metals is likely due to its small grain size, which increases soil contact, especially during traditional threshing methods using cattle hooves. Crop rotation and intercropping also contribute to contamination, as teff is often rotated with onion, bean, chickpea, and lentil, crops that may require fertilizers and pesticides. Residues from these chemicals can remain in the soil, affecting teff quality. To minimize heavy metal accumulation, farmers should avoid rotating or intercropping teff with crops that require high fertilizer use or are not pest resistant.

Although pesticide application is generally uncommon, teff may still be exposed to pesticide residues originating from environmental sources. While the EU has set maximum residue levels (MRLs) to ensure food safety, no such regulations exist in Ethiopia. A study conducted on teff grains from the Jimma Zone detected pesticide residues, including cypermethrin, permethrin, endosulfan, and DDT, at levels exceeding Codex Alimentarius limits by up to 5.6 times [31].

Teff produced in Ethiopia is highly valued for its unique appearance and flavor [39]. The quality of teff is primarily assessed based on its color, which ranges from pale white to ivory white, light tan to dark brown, and even reddish-brown purple. In Ethiopia, grain color is a key indicator of quality and significantly influences market demand and price. The Ethiopian Standard Agency classifies teff grains into four categories based on visual color standards, independent of variety or location of production: (i) very white (‘Magna’, 98–100% white grain), (ii) white (‘Nech’, 95–98% white grain), (iii) brown (‘Key’, 94–100% brown grain), and (iv) mixed (‘Sergegna’, a blend of white and brown grains) [39]. Very white and white teff are the most preferred for food production [16], command the highest prices, and are typically consumed by wealthier households. In contrast, brown teff, although richer in iron, is generally less favored and more affordable, making it accessible to lower-income communities [37,40]. White teff requires more favorable growing conditions and is mostly cultivated in the Ethiopian highlands [31]. Its mild flavor and lighter appearance make it the preferred type, especially among affluent consumers in urban centers such as Addis Ababa, which serves as the largest teff market in the country. The introduction of the improved variety Quncho—meaning “the top” in the local language—has played a significant role in popularizing white teff. Developed by combining traits for high yield and white grain color, Quncho was released in 2006 and became the first improved teff variety widely adopted by Ethiopian farmers, contributing to a 10% increase in productivity [18].

### 3.2. Teff Flour Quality

As already mentioned, teff is a cereal with a small grain size, with a mass nearly 0.7 g/100 g of wheat grain; this makes it difficult to remove its bran. Hence, teff is processed into whole grain flour; as a result, its flour has high fiber content [41]. In fact, due to the tiny size of teff grains, milling does not easily separate the bran and germ, so whole meal teff flour retains substantial amounts of coating layers and sprouts, resulting in high levels of insoluble polysaccharides, essential amino acids, calcium, and iron, which may help explain the low prevalence of anemia in Ethiopian regions where teff is regularly consumed. Teff flour has been studied as a valuable ingredient to improve the quality of gluten-free products in several studies, mainly due to its superior nutritional quality [42]. Experimental evidence also indicates that production origin may influence the compositional and technological properties of teff flour. In a comparative analysis of whole grain teff flours produced in Ethiopia and South Africa, some researchers [41] observed statistically significant differences in most of the chemical, pasting, thermal and functional parameters evaluated. The Ethiopian flour contained 1306 mg/kg of calcium and 363 mg/kg of iron, whereas the South African flour contained 1142 mg/kg and 58 mg/kg, respectively. Conversely, the South African sample showed a higher protein content and contained 34.6 mg/kg of zinc, compared with 24.1 mg/kg in the Ethiopian sample. Because the study examined a limited number of samples and production origins, these values cannot be generalized to all Ethiopian or non-Ethiopian teff flours. Nevertheless, the findings support further investigation of the effects of genotype, environmental conditions and geographical origin on flour composition and functionality [41].

In addition to these compositional and functional aspects, specific quality requirements for teff flour are defined in the Ethiopian Standard ES 3880:2015 [43]. The standard also provides indications relevant to flour quality and storage. The optimum date by which the flour may be used is 8 months from the date of milling; however, once opened, teff flour remains fresh for approximately two months. It is essential to store teff flour in a cool, dark, and dry place, in a sealed airtight container to preserve its freshness and quality. The varieties of teff that can be grown to produce “Ethiopian teff flour” are as follows: Quncho (DZ-Cr-387), Felagot (DZ-Cr-442), Tesfa (DZ-Cr-457), Kora (DZ-Cr-438), Dukem (DZ-Cr-425), and Dagme (DZ-Cr-43B). The above-mentioned varieties can be used individually or blended to produce Ethiopian PGI teff flour. Generally, flour is packaged in bags of 500 g, 1 kg, 5 kg, 10 kg, 25 kg, or 50 kg, and can also be delivered in bulk to any operator [44,45].

## 4. Ethiopian Teff Production System and Value Chain

It is established that economies led by the agricultural sector, such as in the Ethiopian case, should diversify their agricultural products for export to stimulate economic growth. Teff is one of the novel agricultural outputs that can be considered for export since the demand for gluten-free grain products is increasing [27]. Teff is the most widely cultivated cereal in terms of land allocation and ranks second after maize in total production [28,29]. Its popularity is attributed to its low risk in cultivation [18], broad adaptability, and resilience to marginal lands, particularly the heavy, waterlogged clay soils of the highlands where land degradation and population pressure are significant [34].

Teff is indispensable to the livelihoods of many Ethiopians, serving as both a staple food for approximately 70 million people and an important economic asset. It is cultivated by over six million subsistence farmers, who account for most of the country’s agricultural holdings [30,31,34]. These farmers, who generally manage about one hectare per household, account for more than 90% of Ethiopia’s total farm holdings. In 2020, Ethiopia produced 4.4 million tons of teff on 2.7 million hectares, with an average yield of 1.6 metric tons per hectare. As the world’s leading producer, Ethiopia contributes roughly 85% of global teff output. Notably, this growth—averaging 7.4% annually since 2010—has been driven more by the expansion of cultivated land than by technological advancement [31]. Available household-level evidence confirms that total teff production cannot be considered entirely available for commercialization or export. In a study involving 357 teff-producing households across four Ethiopian districts, households consumed, on average, 26.92% of their annual teff production. Average household consumption was estimated at 297.26 kg per year, corresponding to approximately 58.06 kg per person per year for an average household size of 5.12 members [46]. Because these findings refer to selected districts, they should not be interpreted as a nationally representative estimate of domestic consumption or exportable surplus. Nevertheless, they demonstrate that household food requirements must be considered before estimating the quantity of teff potentially available for domestic trade or export.

Teff cultivation is expanding due to rising domestic and international demand for both grain and processed products [22,29]. Moreover, its adaptability across soil types and climatic conditions allows it to be cultivated from lowland to highland regions. Thus, despite its relatively low productivity, teff remains popular because of its cultural relevance and strong market appeal [29]. Evidence from national agricultural surveys also indicates that the Ethiopian teff value chain is undergoing substantial transformation. Based on data covering the period from 2003/2004 to 2016/2017, Bachewe et al. (2019) [47] identified increases in teff production and productivity and showed that cultivated land and labor remained important contributors to production growth, while the contribution of modern inputs and agricultural extension increased over time. Under scenarios incorporating income growth, urbanization and increasing commercialization, the authors estimated that national teff consumption and marketed output could increase by approximately 250% and 300%, respectively, over a 20-year period. These figures are scenario-based projections rather than estimates of future export availability, but they indicate that increasing domestic demand may place considerable pressure on the marketable surplus.

The Ethiopian government is presently encouraging the production of value-added teff products that meet export standards, with the aim of increasing production among small-scale farming households and at the commercial level in all geographical locations of Ethiopia. Teff cultivation clusters are already mapped, and teff producers within the clusters are organized into farmer groups responsible for coordinating the production processes, packaging quality teff grain, and establishing market linkages, often through local farmer cooperatives that partner with higher-level unions and businesses. For milling and flour distribution, small-scale processors are established all over the country. Post-harvest handling represents an important component of this value chain. Using primary data collected from farmers, wholesalers, processors and retailers, Minten et al. (2021) [48] estimated that post-harvest losses along the predominant rural–urban teff marketing pathway amounted to approximately 2.2–3.3% of total production. The authors also found that losses in emerging modern retail systems were approximately half those recorded in traditional retail systems, which they associated with stricter procurement requirements, improved packaging and better storage and sales facilities. Although the magnitude of losses varies according to the stage of the chain and the measurement method adopted, these findings support the need for standardized procedures for threshing, transportation, storage, packaging and handling. Ethiopia is also seeking geographical indications and other entitlements regarding teff and its products to enhance their export potential.

The Ethiopian teff value chain offers an opportunity for improving economic growth and the livelihoods of teff farmers. With appropriate policy instruments to strengthen research and development, input supply systems, and market access, Ethiopia can overcome existing barriers and fully harness the growing global demand for teff. To satisfy both domestic and export demand for teff, the country should continue improving production efficiency by further modernizing farming techniques, providing better access to inputs, including seeds, fertilizers, and farm mechanization equipment, and improving post-harvest storage and milling practices. The existing Farmers Training Centers at each local administrative level, and the agricultural extension system functioning through them, can facilitate farmers’ rapid adoption of new agricultural technologies and best practices to improve teff productivity and grain quality.

At the market level, the Ethiopian government, along with private-sector partners, needs to focus on creating stronger market linkages and value-chain coordination. Promoting the health benefits and peculiar qualities of teff in international markets in Europe and the United States can strengthen Ethiopia’s position as a leading exporter of this gluten-free grain. The value-addition aspect should also be enhanced through branding, obtaining quality certificates, and product diversification, such as various novel teff-blended flours and teff-based food products. These efforts will increase the income of workers throughout the domestic value chain, in addition to increasing export revenues.

To support a future GI application, standardization should cover not only the final teff flour but the entire value chain, from primary production and aggregation to transportation, storage, milling and packaging. Common protocols should address the identity of the varieties and local landraces used, cultivation and harvesting records, threshing practices, moisture and contaminant controls, lot segregation, storage conditions, milling procedures and packaging requirements, as also suggested by studies examining the transformation of the Ethiopian teff value chain and the persistence of post-harvest constraints [47,49].

Teff grain, flour and semi-finished products should be assigned a lot of identifiers linked to the relevant farmer group or cooperative and accompanied by records documenting their geographical origin, handling and transfer along the supply chain. Such measures would help reduce product mixing, adulteration and post-harvest losses, while strengthening food-safety controls and providing the documentary evidence required for end-to-end traceability [30,47,49]. Traceability could also be supported by analytical methods for verifying geographical origin. Studies based on fatty-acid profiles and multi-element fingerprints have demonstrated the potential to discriminate teff grains originating from different Ethiopian production areas [38,39]. However, these analytical approaches require further validation using larger representative sample sets before they can be incorporated into routine control or certification systems.

Standardization should not, however, be interpreted as requiring genetic uniformity. Locally adapted landraces and farmer-managed seed practices could be explicitly recognized within a future product specification, provided that their geographical origin, characteristics and associated production practices are adequately documented. The conservation of landrace diversity is particularly important because Ethiopian teff populations contain substantial genetic and phenotypic variation and are closely linked to farmer-led selection and local adaptation [34,36]. This approach would allow traceability and quality assurance to be strengthened without undermining local genetic diversity, farmers’ rights or traditional agricultural knowledge [50].

At the same time, Ethiopia must take stronger measures to protect its intellectual property rights [20,51] and ensure that the benefits of teff trade remain in the hands of its farmers and local communities. Reviewing and developing updated national strategies and an implementation mechanism for the protection of indigenous knowledge and genetic resources are important to avoid exploitation by outsiders. Collaborative agreements should prioritize partnerships with international organizations that respect Ethiopia’s rights to its agricultural products and indigenous knowledge.

To this end, Ethiopia has already established a comprehensive institutional framework and legislation to protect its genetic resources and traditional knowledge, which are crucial for regulating the commercialization of teff and ensuring that its benefits are retained within the country. These include the Access to Genetic Resources and Community Rights Proclamation No. 482/2006 and the Plant Breeders’ Rights Proclamation No. 1068/2017, supported by the mandate of the Ethiopian Intellectual Property Authority (EIPA). Together, these frameworks provide the country with a system of regulating access, benefit sharing, and the protection of seed varieties. Ethiopia has also joined the World Intellectual Property Organization and adheres to intellectual property conventions such as the Paris Convention and the Berne Convention, which govern patents and copyright [52]. However, gaps in the prevention of biopiracy, such as those previously affecting teff, are still not adequately addressed. These laws will allow Ethiopia to engage in global markets while protecting its indigenous knowledge and natural resources [50,52]. Although Ethiopia already has farmer organizations, production clusters, processing facilities and an institutional framework relevant to intellectual property protection, these elements do not yet constitute an operational GI system for teff. The main outstanding requirements are a collectively agreed product specification, a representative producer organization, harmonized control procedures and end-to-end traceability. The potential value proposition differs across stakeholders: farmers could benefit from collective market access and quality-based product differentiation; processors and traders from clearer standards, improved traceability and stronger product identity; and public authorities from rural development, export diversification and the protection of origin-linked knowledge and genetic resources. However, the realization of these benefits would depend on inclusive governance, effective controls and sustained institutional support [2,53].

## 5. Assessing the Potential for GI Recognition and Policy Recommendations

### 5.1. Assessment of PGI Readiness and Remaining Gaps

From the consumers’ perspective, GIs serve primarily to convey information and ensure product quality. On the producers’ side, GIs play a significant socio-economic role by contributing to rural development and helping to safeguard traditional production methods. In line with EU legislation, GIs can generate positive effects by enhancing a product’s reputation, creating access to premium markets, and allowing producers to command higher prices [8]. This mechanism can be particularly beneficial for small-scale producers in rural areas, offering them a competitive edge against larger, industrialized competitors and supporting the sustainability of local economies. Consumers often perceive GIs as high-value products and are generally willing to pay more for them compared to standard alternatives [9]. As a result, GI products typically achieve higher retail prices. For producers and supply chain actors—particularly those actively engaged in rural development—GIs tend to generate positive outcomes. However, these benefits vary significantly depending on the unique social, economic, and environmental context of the production area. Studies adopting a regional perspective, rather than focusing solely on individual stakeholders, further highlight the potential of GIs as tools for promoting local development and enhancing the economic sustainability of agriculture. Nonetheless, it is important to recognize that the GI label alone does not guarantee the success of a local initiative. Empirical evidence suggests that additional factors—such as strong collaboration among local stakeholders, institutional support, and strategic planning—are essential to fully realize the potential benefits of GI recognition [54]. Table 2 summarizes how the evidence reviewed in this paper maps onto the principal EU PGI requirements and enabling conditions, while also making explicit the main implementation gaps, priority actions, and trade-offs relevant to a future Ethiopian teff flour application.

Some recent publications also suggest that, in developing countries, the expected economic effects of GIs strongly depend on the capacity of producers and other value-chain actors to manage collective reputation, sustain collective action and negotiate the distribution of added value [56,57].

### 5.2. Socio-Economic Opportunities and Trade-Offs

As summarized in Table 2, geographical delimitation represents a key trade-off between strengthening product typicity and maintaining inclusiveness among established teff producers [1]. Another important socio-economic dimension of GIs lies in their potential to generate employment and drive economic growth. In a food market that is increasingly industrialized and standardized, GI labels assure consumers of authenticity, uniqueness, and superior quality [58]. For producers, GIs present an opportunity to differentiate their products and potentially secure higher market prices [59]. By positioning products within a premium segment, GIs can attract investment and foster job creation—particularly in rural areas. These labels establish a direct link between a product and its region of origin, making them a powerful marketing tool that draws consumer attention to specific geographical areas. As these regions are often rural, GIs provide valuable opportunities to support local economies and promote rural development. The protection of GIs can help sustain economic activity and maintain population levels in rural areas, ultimately improving the standard of living for residents. The socio-economic impact of Teff is also evident in its role as a major contributor to Ethiopia’s agriculture, supporting rural livelihoods both directly through cultivation and indirectly through ancillary sectors such as food processing, transportation, and local trade. Rural communities are the main beneficiaries of GI products, particularly in terms of income generation and employment opportunities. In regions where gaining a technological edge is challenging, and advertising costs are often prohibitive for small producers, GIs offer a valuable alternative. They serve as a direct signal to consumers, indicating that the product originates from a specific area and meets certain quality standards. This allows rural producers to differentiate their products in the market without the need for significant investment in technology or marketing [59]. Moreover, the expansion of farmer clusters and cooperatives may facilitate commercialization, collective quality management and access to services. In the literature [55], for example, some authors found that cluster farming may support the commercialization of teff among smallholder farmers. Nevertheless, the long-term economic, social and distributive effects of these arrangements should be monitored to ensure that participation remains accessible to smallholders and that benefits are not concentrated among larger or better-resourced producers. Such support should ensure the effective participation of smallholder producers, and the equitable distribution of benefits generated by commercialization. In particular, standardization, varietal improvement and market development should remain compatible with the conservation of local landraces, farmer-managed seed systems, domestic food-security priorities and the protection of traditional knowledge [34,50].

Despite the potential of PDOs and PGIs, obtaining a clear and consistent picture of their social and economic sustainability impacts remains complex. A study by Bramley (2011) [60] reviewed international literature on the socio-economic effects of GIs, examining their benefits in terms of quality signaling, market access, and rural development. It also identified traditional knowledge and biodiversity as additional goals that the GI system can help promote. While the cited study [60] highlights the promising advantages of GIs, it notes that much of the discussion around their socio-economic benefits remains theoretical due to a lack of concrete empirical evidence. The analysis also warns of the challenges in realizing these benefits, particularly in developing countries. It emphasizes that international GI legislation alone is insufficient to deliver socio-economic gains. Instead, success depends on supportive institutional frameworks and policies that are tailored to the local context. As some authors [60] assert, GIs offer considerable potential benefits. However, achieving these outcomes is neither automatic nor easy—it relies on how the GI system is implemented, enforced, and leveraged. This calls for coordinated efforts from both governments and producers. Developing countries must recognize that the socio-economic outcomes of GIs are highly context-specific, varying by country, region, and product [61]. Therefore, while GIs represent a valuable policy tool, it is crucial for each country to carefully evaluate the potential costs and benefits within their unique environment. Thus, GIs can produce a wide range of socio-economic impacts, but realizing their full potential requires a strategic, context-aware approach.

### 5.3. Production System, Governance and Institutional Readiness

Teff has potential market access and growth in Ethiopia. It plays a key role in meeting household food consumption demands and as a market commodity. The Ethiopian teff value chain aims to increase profitability by enhancing production efficiency and sustainability.

High-yielding teff varieties, farm machinery such as tractors and mounted farm implements, harvesters, threshers, and post-harvest smart storage and milling are being developed by different Ethiopian institutions, notably the EIAR, and Mekelle and Haramaya universities, and the IFPRI [62]. At operational levels, farmers’ associations, cooperatives, unions, agro-dealers, and milling factories are functioning in a coordinated way interlinked with government programs and actively working in the input supply and distribution and teff grain and flour marketing aspects. The Ethiopian farmers are also organized in specialized teff cultivation cluster areas that are identified and mapped as primarily suitable for teff production for market purposes.

Outside Ethiopia, gluten-intolerant people in the West are also favoring increased production of teff [62]. Ethiopia, as a source and main producer of gluten-free teff, sees a big opportunity to integrate it into EU and US markets. In 2005, the Ethiopian government also tried to sign a teff agreement with a Dutch company, Health Performance and Food International, on the Research and Development part in Europe. However, it failed due to a disputed patent over an invention “storing teff for some time increases flour quality and baking” which was already proven in the Ethiopian farmers’ tradition of storing teff for centuries before grinding to get the best baking quality. Teff that has been stored for longer periods of time, say years, receives a higher price in Ethiopia. However, the experiences found on how EPO granted a patent to HPFI laid out procedures Ethiopia may follow to acquire a patent for its genuine and novel teff product [63].

The potential acquisition of GI status for Teff flour under the European Union framework entails significant economic and social implications, particularly for Ethiopia’s rural development and global trade strategy. Economically, GI protection could facilitate Ethiopia’s entry into premium international markets by establishing Teff as a uniquely Ethiopian product, legally protected against misappropriation and patent-related barriers that have previously hindered exports [64]. Such designation enables price differentiation and valorization [65], with Teff already commanding market prices two to three times higher than other cereals like maize. In regions such as Oromiya and Amhara—responsible for nearly 85% of national Teff production [66]—over one-quarter of the output is commercially marketed, supporting regional trade and injecting liquidity into local economies. Teff cultivation plays a central role in agricultural employment and income generation, with its labor-intensive production process—particularly sowing, harvesting, and post-harvest handling—offering substantial seasonal and informal employment opportunities [67]. Women and youth are especially involved in manual tasks, such as harvesting the tiny Teff grains, fostering gender inclusion and social equity [68]. The GI framework may further empower smallholder farmers by formalizing their traditional agricultural knowledge, encouraging equitable benefit-sharing mechanisms, and aligning with Sustainable Development Goals (SDGs), especially SDG 1 (no poverty), SDG 2 (zero hunger), and SDG 3 (good health and wellbeing) [31]. Moreover, the Teff sector contributes directly to household food security, especially in ecologically vulnerable regions like SNNP and Tigray, where its resilience to moisture stress and long shelf-life make it a strategic crop [69,70]. Additionally, GI recognition may foster rural development through increased demand for infrastructure, quality control systems, and institutional capacity to manage certification and enforcement [71]. However, scholars caution that without parallel investments in productivity-enhancing technologies and value chain modernization, GI certification risks preserving low-yield farming systems, limiting broader developmental impacts. Therefore, while GI status presents an opportunity to economically valorize Teff and protect the cultural heritage of Ethiopian farmers, its success hinges on addressing systemic challenges within the Teff economy—ranging from yield gaps to market inefficiencies—through coordinated policy and investment strategies.

### 5.4. Product Specification, Traceability and Regulatory Requirements

Public-sector support will be necessary to translate these scientific and technical requirements into practice. Relevant Ethiopian administrative authorities, working in coordination with the Ethiopian Intellectual Property Authority, agricultural research institutions, standards and food-safety bodies, regional extension services and producer organizations, could support shared laboratory facilities, farmer and processor training, digital or documentary traceability systems, certification costs and the establishment of a representative GI consortium.

Public policies should also facilitate access to suitable harvesting equipment, transportation, storage, milling and processing infrastructure, while strengthening coordination among farmers, cooperatives, processors, researchers and exporters. Previous research has shown that the development of the Ethiopian teff sector depends not only on production increases but also on improved coordination, post-harvest management, input provision and market access [17,18,47].

In addition to what was mentioned before, two other aspects involving a trade-off deserve explicit consideration. First, the geographical delimitation of a future PGI is not merely a technical matter: a narrower area can strengthen the demonstration of the link between the product and the territory, whereas a broader area may foster greater inclusivity but simultaneously weaken distinctiveness and complicate the control system. Second, compliance with geographical indication requirements must remain conceptually distinct from compliance with food safety regulations. Similarly, environmental benefits should be viewed as potential additional advantages rather than automatic consequences of registration; indeed, recent evidence indicates that such benefits are more likely when explicit environmental objectives are integrated into the product specification and the governance system [72].

Defining quality parameters for teff flour can significantly impact its production in Ethiopia. The goal should be to establish a product specification for the geographical indication (GI) protection of this flour, in line with European regulations. Producers and stakeholders interested in this opportunity may form a group/consortium to ensure that the quality characteristics are met. More broadly, this could represent a starting point for Ethiopian teff flour producers with two main objectives: (i) the alignment and sharing of quality standards among small and medium local producers; (ii) boosting worldwide awareness of a flour with high export potential (e.g., high protein content, gluten-free).

Further development of the Ethiopian teff sector should be supported by a coordinated scientific research agenda involving Ethiopian and international universities, research institutes, public-health organizations and food-sector stakeholders. Teff is well characterized as a naturally gluten-free whole grain containing dietary fiber, proteins, minerals and several potentially bioactive compounds [16,31,73]. Its composition and functional properties also make it suitable for different gluten-free food applications [41,42]. Nevertheless, the evidence supporting specific physiological and health effects remains heterogeneous and is frequently based on compositional analyses, in vitro experiments, or a limited number of studies involving human participants [31,74]. For example, a study [75] examined the inclusion of teff in the diets of people with coeliac disease, but the currently available clinical evidence remains insufficient to support broad or product-specific health claims.

### 5.5. Research Priorities and Market Development

Future research should therefore investigate how genotype, local landraces, geographical origin, soil and climatic conditions, storage and processing influence the nutritional composition and functional properties of teff. Particular attention should be given to nutrient bioavailability, protein and starch digestibility, glycemic response, the effects of fermentation and milling, and longer-term health outcomes in relevant population groups [31,73,74]. A stronger evidence base would support scientifically responsible communication of teff’s nutritional characteristics, facilitate the development of innovative teff-based products and help identify measurable attributes that could potentially be included in a future PGI product specification.

## 6. Conclusions

This review assesses the extent to which scientific, regulatory, institutional and socio-economic evidence supports the recognition of Ethiopian teff flour under the EU Protected Geographical Indication (PGI) scheme. The findings indicate that Ethiopian teff possesses several PGI-consistent characteristics. In particular, its long-standing geographical, cultural and socio-economic connection with Ethiopia, distinctive agro-ecological characteristics, differentiated production areas, established cultural reputation, growing international relevance and emerging analytical evidence for geographical authentication collectively support its potential for recognition under the EU GI framework. However, critical gaps currently preclude a fully operational application, notably the absence of a legally protected national GI framework, an agreed geographical delimitation defining the appropriate extent of the production area, a collectively agreed product specification, representative producer governance, harmonized control procedures and end-to-end traceability systems.

The available evidence points to a strategic opportunity to enhance the visibility, quality assurance and market potential of Ethiopian teff, a culturally and nutritionally significant crop; however, translating this evidence base into an operational PGI requires addressing several implementation conditions. Going forward, three complementary dimensions should be considered: (i) consolidation of eligibility (i.e., translating the substantial existing evidence on the product-origin link into a precisely defined geographical area and a legally robust product specification); (ii) readiness (i.e., ensuring that the required governance, traceability, quality control and national legal protection are fully implemented and effective); (iii) desirability (i.e., assessing whether the expected benefits justify the compliance costs and possible distributive, food-security and biodiversity trade-offs).

Advancing this long-term process will require the convergence of scientific action, producer-led initiatives, and public sector intervention. Universities and research centers can continue to provide the scientific evidence and technical support needed to strengthen production specifications and control systems, while producer organizations and other forms of collective cooperation should progressively emerge and consolidate through a bottom-up process rooted in the production system. Particular attention must be paid to the meaningful involvement of small-scale producers, both women and men, who should be among the primary beneficiaries of the economic, social, and territorial value generated by GI recognition.

A genuinely bottom-up process should foster participation and ownership, equitable access to decision-making and benefits, a shared culture of quality, stronger organizational capacity and greater entrepreneurial skills among small-scale producers, regardless of gender, and local value-chain actors. At the same time, the Ethiopian government has a crucial enabling role in supporting this process through sustained public investment, institutional coordination, capacity building, extension and advisory services, and measures facilitating producer organization and market development. The combination of scientific expertise, inclusive producer-led collective action and strong public-sector facilitation could therefore transform the existing evidence and local potential into an operational and durable GI pathway of strategic importance for Ethiopia. If developed under these conditions, PGI recognition could contribute to the valorization of Ethiopian teff, strengthen rural economies and provide a relevant example for other origin-linked African products, while recognizing that registration alone cannot guarantee rural-development or sustainability outcomes.

## Figures and Tables

**Table 1:** Specific characteristics of PDO and PGI certifications.

	PDO	PGI
**Link with geographical origin**	Quality or characteristics essentially or exclusively due to the geographical environment	Quality, reputation, or other characteristic essentially attributable to geographical origin
**Production stages**	All production steps within the defined geographical area	At least one production step within the defined geographical area
**Emphasis**	Geographical environment and its inherent natural and human factors	Geographical origin

**Table 2:** Comparison between EU PGI requirements and enabling conditions, the evidence available for Ethiopian teff, and the principal gaps, priority actions, and trade-offs for future PGI development.

EU PGI Requirement/Enabling Condition	Evidence or Enabling Conditions for Ethiopian Teff	Current Gap, Priority Action, and Main Trade-Off
**Theme 2A. Product identity, provenance and scientific evidence**		
Link with geographical origin and defined area	Ethiopia is the center of origin and diversity of teff; long-standing cultivation, local landraces, differentiated production zones, and preliminary fatty-acid and multi-element markers provide origin-related evidence [21,34,38,39].	A proposed geographical area must be delimited and the product–origin link substantiated across varieties, harvest years, and environments. A narrow area may strengthen typicity but could exclude established producers outside the boundary.
Product reputation and distinctive characteristics	Teff is a national staple with strong cultural heritage; market differentiation by color and production area and the reputation of specific origins such as Bichena provide relevant evidence [27,37,38,39,40].	Historical, market, and documentary evidence should connect the proposed name and reputation specifically to the defined area. Nutritional or health attributes should not be used as origin claims unless adequately substantiated.
Scientific substantiation and geographical authentication	Studies report variation in composition and functionality and show promising origin-related compositional/functional differences or discrimination by fatty acid and multi-element profiles [38,39,41].	Multi-year, geographically representative studies are needed to disentangle genotype, environment, and origin effects and to validate authentication methods before certification. Evidence for specific health effects also remains insufficient for broad claims.
**Theme 2B. Product specification, production and control system**		
Product specification and single document	Ethiopian Standard ES 3880:2015 provides a basis for flour quality requirements, complemented by information on varieties, storage, and packaging [43,44,45].	A complete PGI specification is still needed, defining the name, product characteristics, geographical area, eligible varieties/landraces, production steps, origin link, labelling, packaging, and controls. Standardization should remain compatible with genetic diversity and farmer-managed seed systems.
Standardized production and post-harvest controls	Extension services, farmer clusters, cooperatives, milling facilities, and evidence on post-harvest loss points provide an operational base [47,48,49].	Harmonized protocols are needed for cultivation records, harvesting, threshing, transport, storage, milling, and packaging. Modernization should improve safety and consistency without eliminating traditional practices that form part of product identity.
Traceability and chain of custody	Farmer clusters, cooperatives, processors, and the proposed use of lot identifiers provide a starting point for documentary traceability [47,48,49].	End-to-end records are needed from farm/seed source through aggregation, storage, milling, packaging, and transfer. Segregation and documentation will add compliance costs that must remain proportionate for small producers.
Verification of compliance and food-safety controls	National standards and existing research identify relevant quality and safety parameters, including mycotoxins, pesticide residues, and other contaminants [31,43].	The future specification requires a credible verification system and routine testing capacity. Independent certification/control arrangements, laboratory capacity, and their cost are major implementation constraints. A PGI control system can enhance traceability and quality management, but it does not replace the requirements regarding contaminants, residues, hygiene, and imports applicable to food placed on the EU market.
**Theme 2C. Regulatory and governance requirements**		
Legal protection in the country of origin and third-country application route	EIPA, the GOPE initiative, and the 2025 final draft of a national GI law demonstrate institutional progress toward a dedicated Ethiopian GI framework [10,11,12,13].	For a third-country GI, EU law requires legal proof of protection in the country of origin [4]. Domestic protection of the proposed teff GI and an agreed filing route—directly by an applicant producer group or through the competent Ethiopian authority—are therefore prerequisites.
Applicant producer group and governance	Existing cooperatives, unions, and farmer production clusters provide organizational building blocks [47,55].	A representative producer group with democratic governance must be established or designated for the application and subsequent GI management. Participation and decision-making should remain accessible to smallholders and avoid concentration of benefits.
**Theme 2D. Socio-economic enabling conditions and safeguards**		
Public governance, market impacts, and socio-economic safeguards	EIPA, research institutions, extension services, producer organizations, and existing market structures provide institutional assets; teff also has major domestic food-security and livelihood importance [46,47,52,55].	Policy support is needed for laboratories, certification, extension, traceability, and market development. GI development should protect domestic food-security priorities, local landraces, farmers’ rights, and equitable benefit sharing while avoiding exclusion of less-resourced producers.
Domestic food security and marketable surplus	Teff is a staple food for approximately 70 million Ethiopians and is cultivated by more than six million smallholder farmers. Household-level evidence also indicates that a substantial share of production is retained for domestic consumption, while projected growth in domestic demand may place pressure on the marketable surplus [30,31,46,47].	The scale of genuinely exportable surplus should be assessed before GI-driven market expansion. Export promotion should not compromise domestic food availability; monitoring of production, household consumption and marketed volumes is therefore required.
Landrace conservation, farmers’ rights and equitable participation	Ethiopian teff is characterized by substantial landrace diversity, farmer-led selection and locally adapted seed systems. Existing farmer groups and cooperatives provide a basis for collective participation [34,47,50,55].	Standardization and varietal requirements should preserve local genetic diversity and farmer-managed seed systems. GI governance should ensure meaningful participation of smallholders and equitable access to decision-making and benefits.

## Data Availability

No new data were created or analyzed in this study. Data sharing is not applicable to this article.

## Abbreviations

The following abbreviations are used in this manuscript:

GI	Geographical Indications
PGI	Protected Geographical Indication
PDO	Protected Designation of Origin
TSG	Traditional Specialty Guaranteed
EPO	European Patent Office
EU	European Union
TRIPS	Trade-Related Aspects of Intellectual Property Rights
WTO	World Trade Organization
AFB1	aflatoxin B1
OTA	ochratoxin A
MRLs	maximum residue levels
SNNP	Southern Nations, Nationalities, and Peoples
FAO	Food and Agriculture Organization
SDGs	Sustainable Development Goals
EIAR	Ethiopian Institute of Agricultural Research
IFPRI	International Food Policy Research Institute
HPFI	Health and Performance Food International
EIPA	Ethiopian Intellectual Property Authority
